# Students’ Perceptions of Peer-Organized Extra-Curricular Research Course during Medical School: A Qualitative Study

**DOI:** 10.1371/journal.pone.0119375

**Published:** 2015-03-12

**Authors:** Bassel Nazha, Rony H. Salloum, Akl C. Fahed, Mona Nabulsi

**Affiliations:** 1 Department of Internal Medicine, NorthShore LIJ Staten Island University Hospital, Staten Island, NY, United States of America; 2 Department of Neurosciences, Lerner Research Institute, Cleveland Clinic, Cleveland, OH, United States of America; 3 Department of Internal Medicine, Massachusetts General Hospital, Boston, MA, United States of America; 4 Department of Pediatrics and Adolescent Medicine, Faculty of Medicine, American University of Beirut, Beirut, Lebanon; University of Torino, ITALY

## Abstract

Early integration of research education into medical curricula is crucial for evidence-based practice. Yet, many medical students are graduating with no research experience due to the lack of such integration in their medical school programs. The purpose of this study was to explore the impact of a peer-organized, extra-curricular research methodology course on the attitudes of medical students towards research and future academic careers. Twenty one medical students who participated in a peer-organized research course were enrolled in three focus group discussions to explore their experiences, perceptions and attitudes towards research after the course. Discussions were conducted using a semi-structured interview guide, and were transcribed and thematically analyzed for major and minor themes identification. Our findings indicate that students’ perceptions of research changed after the course from being difficult initially to becoming possible. Participants felt that their research skills and critical thinking were enhanced and that they would develop research proposals and abstracts successfully. Students praised the peer-assisted teaching approach as being successful in enhancing the learning environment and filling the curricular gap. In conclusion, peer-organized extra-curricular research courses may be a useful option to promote research interest and skills of medical students when gaps in research education in medical curricula exist.

## Introduction

Medical students’ perceptions and conceptualizations of medical education and their learning environment may impact the quality of their training, and the paths they choose for their future careers [1]. One of the reasons why fewer medical students choose academic tracks is the lack of exposure to research during undergraduate medical education [2]. Despite the fact that medical students understood the value of research, many were oblivious to research projects being undertaken within their own university, and tended to have a limited understanding of what research entailed [3]. A survey of 932 medical students who attended a course on research methodology during their second year revealed that attendance of the course was related to a positive attitude towards science among students [4]. However, attitudinal change towards research may not necessarily reflect the actual behaviour of students or later engagement in research conduct. A review of the post-graduation scientific output or publications of 274 medical students showed that those who engaged in extra-curricular research experiences during medical school had a significantly greater research output than their peers after graduation [5]. These findings highlight the importance of integrating research opportunities early into medical curricula. Yet such opportunities may not be easily accessible to medical students, as many medical schools lack an integrated research component in their curricula [2].

It has been argued that allowing medical students a voice in the development of their curriculum is important for effective clinical training [1, 6]. As such, a group of medical students at the American University of Beirut (AUB) decided in 2008 to fill the research education gap in their medical curriculum by organizing their own yearly research course to help them build research skills. The Medical School at AUB follows the American model of medical education with a 4-year curriculum, and the language of instruction is English. Though its Medical Center has ongoing basic and clinical research projects, the School does not require a research thesis for graduation, and the vast majority of students are not involved in research. Although students get exposed to Epidemiology and Biostatistics courses during their preclinical years, formal training in research methodology is limited to a short 2-week, team-based research activity as part of Social and Preventive Medicine course during the first year of Medical School.

While attitudinal changes are critical for behaviour modification at the individual level, so is the creation of a facilitating environment for this behavioural change to happen. With this background in mind, we aimed in this study to explore the experiences of medical students at AUB who participated in the fourth research course (the facilitating environment) that was organized by their peers during the academic year of 2011–2012, and to investigate whether this unique extra-curricular academic activity changed their perceptions and attitudes towards research, including their choices of future careers.

## Materials and Methods

### Ethics Statement

This study was approved by the Institutional Review Board of the American University of Beirut. Each participant signed a written informed consent and gave permission to tape-record the discussion. Participants were aware of the nature and objectives of study. Participants were also assured confidentiality and anonymity of recordings, transcripts and any behaviors observed during discussions; the voluntariness of participation and withdrawal, and that gathered information would be used solely for the purpose of the study.

### Course Description

Following the success of the first research course in 2008, it became a solid extra-curricular activity organized yearly by medical students at AUB, attracting many of their peers who are interested in research training. The fourth course, subject of this study, was conducted between December 2011 and April 2012 and consisted of 14 sessions covering all steps of the research process. Sessions were moderated mostly by volunteering faculty members who were recruited by the organizing team of medical students. Student organizers who previously attended similar courses also moderated some sessions of the course (Table 1). The specific roles of student organizers and faculty are outlined in Table 2.

**Table 1:** List of course topics.

	Session Title	Moderator
1.	Introduction to research	Student
2.	Overview of the research process	Student
3.	Formulating the research question & Developing a working hypothesis	Faculty
4.	Conducting an effective literature review	Faculty
5.	Round-table discussion: From basic to clinical research	Faculty & Student
6.	Study designs: Cross-sectional and case-control studies	Faculty
7.	Study designs: Cohort studies and clinical trials	Faculty
8.	Data collection, entry, cleaning and management	Faculty
9.	Descriptive and inferential data analysis	Faculty
10.	Round-table discussion: Research dissemination	Faculty & Student
11.	Research ethics	Faculty
12.	Funding opportunities	Faculty
13.	IRB regulations and processes	Faculty
14.	Research Day presentations	Students
15.	Setting long-term plans for projects and feedback	Faculty & Students

**Table 2:** Roles of student organizers and faculty in the research course.

Student Organizers	Faculty
Two students (fourth year) co-coordinated the course with a faculty member	One faculty member co-coordinated the course with the student co-coordinators
Two students (second and third year) assisted the course co-coordinators	Selected faculty by the course coordinators were invited to assist in moderating sessions
Selected students who attended the course in previous years moderated sessions	Selected faculty by the course coordinators served as mentors
Students who previously attended the course (2008–2011) and were in postdoc positions after graduation served as mentors for students enrolled in course	Faculty co-coordinator and Associate Dean for Research mentored student organizers and approved their final syllabus of the course
Developed the syllabus of the course	Faculty co-coordinator assisted students in recruiting faculty moderators and mentors to the course
Selected faculty members and fellow students to moderate particular sessions	Provided full funding for the course
Approved the delivery method of each session with emphasis on interactive sessions and hands-on experiences	Interviewed applicants and decided on admissions to the course (committee: 2 faculty members and one student)
Interviewed applicants and decided on admissions to the course (committee: 2 faculty members and one student)	
Managed all logistics (course material, mailing list, activities, homework, etc.)	
Organized the Research Day, a university-wide activity, in which students from the course were given priority to present their work	
Worked closely with students to ensure healthy mentor-mentee relationships throughout the year and progress on the different research projects	

The course educational tools varied between didactic lectures, round table discussions, mock presentations, and hands-on tutorials. Each participant student was required to choose a research question and a mentor who would guide him/her through the process of developing the question into a full research proposal. Moreover, students were offered lists of recommended readings, useful links, mentors and a database of ongoing research projects at AUB. At the end of the course, students presented abstracts of their research proposals in the Research Day, a yearly activity during which students and residents present their research projects.

### Study design

All 26 participants of the research course (8% of the total medical student body) received an email by BN four months after the end of the course to participate in one of three focus group discussions, each consisting of 5–8 students. Focus group discussions are effective data collection tools used in qualitative research to explore participants’ feelings, perceptions or experiences towards a certain topic of interest [7, 8]. We used an interview-guide consisting of 7 open-ended questions about previous experience in research, perceptions towards research prior to the course and afterwards, strengths and limitations of the course, and the effect of this experience on the choice of future career path (S1 Appendix). The guide was developed in light of students’ comments and feedback that was provided systematically after each session of the course. The focus group discussions were conducted in English by a hired female facilitator who holds a Masters in Public Health (MPH), and who is experienced in qualitative research. The facilitator introduced her background to participants during the focus groups as she was previously not involved with the students in the course. Each focus group took place in a conference room at AUB and lasted for one hour. Only the facilitator and the participants were present. The former took field notes during the focus groups. All discussions were taped, transcribed in verbatim, coded and analysed by the facilitator using inductive thematic analysis. Recurrent themes emerging from raw data were identified and coded, and major and minor themes with similar codes were summarized on spreadsheets to provide insight into students’ experiences and feelings. The transcripts and generated themes were shared only with the authors who reviewed all transcripts a second time to minimize any facilitator’s bias, and checked the generated themes to validate the findings. The findings were not shared with the participants for feedback.

## Results

Of the 26 medical students who participated in the course, 21 (12 females) consented to the focus group discussions. The majority of participants were in preclinical years (13 in first year, 2 in second year), with only 6 in clinical third year. For the sake of this paper, participants were assigned random numbers presented along with their corresponding medical year. Several themes of interest regarding students’ perceptions were generated from the group discussions. These included the following: research being difficult for students; change of students’ attitudes towards research; enhancement of research skills; development of critical thinking; improvement of writing skills; impact of mentor-mentee relationship on students’ experiences; value of peer organization; additional course achievements; course limitations (S1 Table).

### Research is difficult for students

Most participants were not exposed to research prior to the course with only two having had modest laboratory research experiences. Students perceived conducting research to be difficult at their level since they lacked the necessary clinical experience needed to generate research questions. They felt that research is complicated and takes time to accomplish, which a first year student described as a “long continuous process” that is “time consuming” and “only doctors can do”:

“it seems too complicated to be conducted by ourselves independently. We do not have the courage to go ahead and lead a project”. (First year 9)

Yet, students were driven by their curiosity to join this course to learn more about the research process, starting with their own research questions.

### Change of students’ attitudes towards research

Most students reported that following this course, they developed a new appreciation for research. They realized that research and medicine are complementary to each other, and that research involves other disciplines as well like Public Health and Basic Sciences. Moreover, their previous perceptions that research would be difficult for students changed, and they felt it became possible after this course, as well as being relevant to clinical practice:

“At first, I thought we should be doctors and have ten years of practice before we could go into research, but I learned that I can start research now”. (First year 7)

One first year student reflected on the guiding impact of research on physicians’ clinical decision-making very much similar to an evidence-based practitioner, even though she was not exposed yet to evidence-based medicine:

“I think research would help me as a physician. knowing what treatment is adequate for the patient, the advantages and disadvantages of the treatment according to research, so it strengthens your medical career”. (Third year 4)

This positive change in students’ attitudes towards research encouraged some students to engage in clinical research in the future, and motivated others to consider pursuing a career in research after graduation.

### Enhancement of perceived research skills, critical thinking and writing skills

The majority of participants elaborated on the research skills they gained from the course, and how they could systematically reproduce the stepwise process of designing a research project. They realized the importance of phrasing a well-refined research question and conducting an effective literature search. They valued the fact that their analyses of research papers became more critical, and that the course improved their writing skills. This is best reflected by the following quote of a first year student describing how she could replicate the research process in the Social and Preventive Medicine course, which she took after the research course:

“It was the final year project of the SPM course, and we had to come up with a full research idea, submit to IRB, do data collection, analysis and write-up in 2 weeks! If I hadn’t gone through this experience (in the course) it would have been a disaster!” (First Year 2)

Another third year student reflected on how the course transformed the way she reads or interprets research papers, and how it shaped her writing skills:

“Now when I read a research article I read it differently. I always skipped the methods section. Now I know it’s actually more important than the results!” (Third Year 2)

### Impact of mentor-mentee relationships on students’ experiences

Most students appreciated sharing their research experience with professors who volunteered to lecture or mentor them. Whether lecturers in sessions or mentors on projects, faculty participation was valued by students for the lifetime experiences they provided. The course provided the opportunity for students to build personal connections with faculty that otherwise would not have been easily established. The mentor-mentee relationship affected the students’ experiences and their expectations both positively and negatively. Whereas some students had supportive mentors, others had little guidance from their mentors, or had a hard time finding a mentor who shared the same research interest. Some students had to change their research questions in order to match their mentors’ interests:

“I think what’s good about it, other than giving us lectures about research in general, is the round-table discussions we had. They were very useful because the professors got a chance to tell us more about their subjective views of research: if you end up as a clinician or in basic sciences, how you can pursue research, how it affects your life, what are the benefits.” (First year 6)

“I had a mentor but he was not helpful. I wrote the entire proposal alone. There was no feedback, and at the end, he did some modifications and submitted to a committee”. (Second year 1)

It is interesting to note that the course alerted the students to the importance of discussing authorship rights and work expectations with their mentors when planning the research project, as well as the duties and expectations from each party.

### The value of peer organization of the course

Participants valued the fact that the course was organized by their peers. Participation in the course was motivated by personal interest and self-fulfillment with no pressure for grade. In that context, student organizers were perceived by their peers to be facilitators of learning. They were seen as very helpful in guiding their peers to appropriate mentors, and were available for advice and feedback on regular basis:

“I like the fact that it was organized by students. because when we needed help and could not go to the doctors [mentors] for basic stuff. we could always turn to them [peers] for help”. (First year 1).

### Additional course achievements

Most students felt that their expectations from the course were met as they succeeded in developing their research proposals, and hence bridged the gap in their medical curriculum regarding research education. However, this journey was not without difficulties. Students for example, were expected to submit assignments after every session to course coordinators and/or their mentors relating to their projects. Such deadlines were viewed by some students as stressful, leaving them with little time to change research questions if they had to, or to accomplish assignments by the deadline. Yet, most students reported that having deadlines prompted them to work more efficiently and hence enhanced their time management skills. Moreover, the deadlines helped them succeed in reaching the Research Day with research abstracts that summarize their questions and methods of inquiry, and are suitable for presentation in that forum:

“Developing our proposal made the whole research course coherent, important, and concrete.”(First year 12)

“This was like a motive for us to show others what we’ve been working on for several months”. (Second year 2)

### Course limitations

Students reported several course limitations and suggested improvements for future courses. These included difficulty finding a mentor, setting tight deadlines, the didactic nature of some sessions, and the heterogeneity of participants in terms of background knowledge and clinical experience. Preclinical students for example found it difficult to come up with research ideas since they lacked the clinical exposure that will help them find research questions, as compared to students in clinical years. On the other hand, biostatistics and epidemiology lectures were “incomprehensible” for preclinical students and “repetitious and boring” for students in clinical years.

These limitations prompted students to suggest improvements such as enhancing the engagement of mentors, replacing didactic lectures with interactive sessions, and dividing participants into small groups with diverse backgrounds thus allowing students to teach each other:

“concerning the diversity of student population, Medicine 1, 2 and 3, I think there are ways to go around this limitation with small working groups that have students from different classes. The more senior students can help the juniors grasp the info better than having a lecture that is advanced for some and trivial for others”. (Third year 3)

The generated themes were very much in line with the feedback previously obtained from students at the end of each session during the course. Student ratings of most sessions ranged between “Good” to “Excellent”, as was their overall assessment of the course. For example, the data collection and management session was judged to be “Excellent” by 9 students and “Good” by 7. Suggested improvements in the evaluations from that session included the need for hands-on exercises and work in small groups in order to better grasp the explained concepts. Similarly, two aspects of the course sessions that students frequently valued were the feedback they received individually on their on-going projects, the active interaction with the moderators, and the practical skills they were learning. Interestingly, sessions in which a student organizer was presenting were remarkably well received, such as the one on research dissemination, which was rated as “Excellent” by all 17 students who submitted their forms, a finding that attests to the value of peer organization.

It is interesting to note that, one year after the course, 12 of the 26 (46%) attendees of the research course continued to be involved in research, with 8 of 11 (73%) students in clinical years then participating in research projects. All five students who served as course co-coordinators from 2008 through 2012 pursued further research training (2 postdoctoral, 2 PhD, 1 MPH) after graduation from medical school.

## Discussion

Research education and training during medical school is essential to identify physicians-in-training who may pursue a career in academic medicine later. Evidence reveals that students who participate in research during medical school publish significantly more articles during their postgraduate training [5, 9]. Our data supports that research education and training is possible in the early years of medical school. Such training did not only impact students’ attitudes toward research positively, but also impacted their perception of self-efficacy in conducting research and writing research proposals and abstracts. Moreover, early involvement in research may enhance students’ critical thinking and their appreciation of the strong link between research, clinical practice and evidence-based medicine. These findings are in line with the Theory of Reasoned Action and Theory of Planned behaviour [10]. Both theories assume that “attitude toward certain behaviour and social normative perceptions determine behavioural intention and thus best predict such behaviour” [10]. Interestingly, in a mixed method study on required research electives at UK medical schools, students statements on the benefits of their developed research skills were very similar to ones reported by our participants: ‘‘it’s the development of a skill that you’re going to have for the rest of your life” [11].

Medical students’ development of research skills may however be hindered by several barriers such as time constraints, lack of curricular requirement of research training in most medical schools [12], and negative mentorship experiences. Most of these barriers were cited by our participants as the major barriers that affected their research experience during the course negatively. Such barriers need to be thought of when designing similar research methodology courses in the future, so as to maximize students’ benefits from such courses.

To the best of our knowledge, this study is the first one to shed light on the credibility of peer-assisted learning of research methodology in medical school. The value of peer-assisted learning lies mostly in it being done out of personal interest, a need for self-fulfilment, and being free from pressure for grade. Peer-assisted learning allows participating students to receive education tailored to their cognitive level thus enhancing their motivation to learn. Furthermore, it prepares organizing students for their future roles as physician educators [13, 14]. Although the literature lacks reports on extra-curricular student activities targeting research education outside of the medical curricula, there is evidence that medical students’ extra-curricular activities in the community, such as initiation of programmes targeting sexual violence among youth, or decreasing the white-coat fear in small children (known as Teddy bear hospital), are indeed successful. In some hospitals, the success of such activities led to their integration in the official curriculum. Moreover, the partnership between faculty and students in such programmes was reported to positively impact the student-faculty relationship, thus enhancing the learning experience of students [15]. We believe that the peer-assisted learning aspect of our course strongly contributed to its success.

Using Miller’s educational model [16] as a systematic framework for evaluating the impact of this course, one may argue that students, who after the course perceived themselves as competent (know how) in conducting research, have moved a step further in performing (showing how), by continuing to be actively engaged in basic or clinical research. Determining whether the highest level of Miller’s model (doing independently) will be reached requires a quantitative assessment of the research productivity of all students who participated in the course since it started. Such analysis, however, was beyond the scope of our study.

We chose to conduct focus group discussions because qualitative methods are best suited to capture the experiences, perceptions and attitudes of students. In this study, we used purposive sampling and invited all students who participated in the research course to enrol in the focus group discussions, in order to capture most of their experiences and perceptions. Only five students declined as they were outside the country when the study was being conducted. Although the major themes were recurring in all transcripts, we cannot assume that saturation was reached. To ensure validity of findings, three of the authors reviewed all transcripts, major and minor themes, and found them to be consistent with those of the facilitator who conducted the analysis.

Our study has some limitations. Because of its qualitative nature, our findings may not be generalizable to other medical schools since all participants belonged to the same institution. Given the limited number of participants in the course, one may make a case that the receptivity to the course might differ if it was implemented to a larger group within the same institution. However, the purpose of the focus group discussions is to explore the depth of the findings rather than their breadth, or generalizability. Another limitation is the fact that attitudinal change may not translate into behavioural change later. As previously mentioned, assessment of the long-term effects of this course on participants’ future research productivity is necessary. Also, since the participants were self-selected, it may be argued that the success of the course could be due to highly motivated students who were eager to have research training early in their medical education. Despite these limitations, we believe that this extra-curricular activity can be a good model to replicate by other schools with similar educational needs.

## Conclusions

A peer-organized extracurricular research methodology course may be a useful resource to train medical students in research skills, and can fill gaps in medical curricula. Such courses can positively change students’ attitudes towards research and may potentially change their behavior and encourage them to engage in research early on. Moreover, having research courses as core curriculum is essential for identifying future clinician researchers. Further studies in similar settings are needed to confirm our findings.

## Supporting Information

S1 Appendix Focus group discussion interview guide. (DOCX)

S1 Table Themes and reflective quotes generated from transcripts. (DOCX)
