# Supplementary material for: Students’ Perceptions of Peer-Organized Extra-Curricular Research Course during Medical School: A Qualitative Study
Source: PLoS One. 2015 Mar 12;10(3):e0119375. doi: 10.1371/journal.pone.0119375 (PMC4357456; doi:10.1371/journal.pone.0119375)
Supplement: S1 Appendix — (DOCX) [file pone.0119375.s001.docx]

**S1 Appendix. Focus group discussion interview guide.**

1. What can you tell me about your experience in the research workshop?
2. What were your perceptions of research prior to this course?
   1. What were your feelings about the importance of research?
   2. What was your previous involvement in research?
   3. What were the difficulties you faced when conducting research?
   4. How was your experience with your mentor?
   5. What can you tell me about research grant application?
3. How do you think this course changed your perceptions/attitudes towards research?
4. What are the strengths of this course/experience?
5. What were the limitations of this course/experience?
6. How do you think this experience has shaped your future career options?
   1. Conducting research in the future
   2. Choosing an academic career/clinical career
7. Do you have anything more to add?
