# Supplementary material for: Students’ Perceptions of Peer-Organized Extra-Curricular Research Course during Medical School: A Qualitative Study
Source: PLoS One. 2015 Mar 12;10(3):e0119375. doi: 10.1371/journal.pone.0119375 (PMC4357456; doi:10.1371/journal.pone.0119375)
Supplement: S1 Table — (DOCX) [file pone.0119375.s002.docx]

**S1 Table. Themes and reflective quotes generated from transcripts.**

| **Themes** | **Reflective Quotes** |
| --- | --- |
| Research is difficult for students. | *-“it seems too complicated to be conducted by ourselves independently. We do not have the courage to go ahead and lead a project”. (First Year 9)*  *-“I didn’t know much about research. That’s why I felt I needed guidance and thought the course would be a good way to start”. (First Year 3)*  *-“I didn’t know much about research. That’s why I felt I needed guidance and thought the course would be a good way to start”. (Second Year 2)* |
| Change of students’ attitudes towards research.  Enhancement of perceived research skills, critical thinking, and writing skills. | *-“.. research is not separate from Medicine, it complements it”. (Frist Year 13)*  *-“I realized there is the clinical, the public health research, not only lab..” (First Year 8)*  **-“***I think research would help me as a physician.. knowing what treatment is adequate for the patient, the advantages and disadvantages of the treatment according to research, so it strengthens your medical career!” (Third Year 4)*  *-“..research is a plus in medicine because..you get to investigate something you’re interested in and come up with your own observations..I thought research would be boring but this completely changed now”. (Third Year 5)*  *-“At first, I thought we should be doctors and have ten years of practice before we could go into research, but I learned that I can start research now”. (First Year 7)*  **-“**.. *I am really considering having a career in research as well as clinical practice at the same time”. (First Year 2)*  *-“The course gave structure on how to conduct research and follow it up”. (Third Year 6)*  *-“..the most important skill is how to come up with a research question and refine it...” (First Year 5)*  *-“..even the simplest idea in the world is enough..to have a very clear hypothesis..in a very clear research question!” (First Year 10)*  **-**“..*we have all the databases, journals..but we didn’t know how to search them..how to filter them. This was one of the skills we learned ..how to get what we want from the database”. (First Year 4)* |
|  | **-“***Now when I read a research article I read it differently..I always skipped the methods section. Now I know it’s actually more important than the results!”(Third Year 2)* |
|  | *-“This is not something we learned in medical school..how to write an introduction..methods”. (Third Year 1)*  *-* “*..you know now how to write a good abstract that grabs attention, is concise and interesting..” (First Year 11)* |
|  |  |
| Impact of mentor-mentee relationship on student’s experiences. | *-“..we built personal connections with well-placed researchers and gained ever-lasting tools that we can use in the future in our medical practice or research”; “..with every session we had a different doctor, and each spoke of his research experience, so this like summarizes 20 years of his experience..” (First Year 11)*  *-“Working with my mentor was the most inspiring part”. (First Year 6)*  *-“The best thing was when my abstract was corrected by my mentor, I saw the difference..lots of experience [gained] for future abstracts..”  (First Year 12)*  -“*it is difficult to actually find a mentor.. because everyone has an idea that falls in a different field [of interest]”*. *(Third Year 6)*  “*..students need their mentors to teach them but the mentor needs the student also to help him in his research. Sometimes it is free voluntary work. But you need to agree ahead of time. Like I’m going to work on this research, what am I going to get out of it, before the work with the mentor begins!” (First Year 8)* |
| Value of peer organization. | *-“He [student organizer] taught me how to deal with my mentor and what to expect from him”. (First Year 7)*  *-“..it was a very dynamic learning experience..even the student coordinators would give us feedback sometimes..and this would motivate us because they are like us: medical students..friends..yes, the peer effect!”(First Year 5)*  *-*“*I like the fact that it was organized by students..because when we needed help and could not go to the doctors [mentors] for basic stuff..we could always turn to them [peers] for help”. (First Year 1)* |
| Additional course achievements.  Course limitations. | -“*It filled the gaps in our curriculum that does not stress research”.  (Second Year 2)*  *-“If we had not done the exercise [of developing the proposal], we would not ..realize all the work it needs”. (First Year 3)*  *-“They forced us to find an idea and to work on it. I think it was somehow beneficial to push us to do the work in a 2-week period. If they did not put deadlines, we would have stopped”. (Third Year 2)*  -*“.. in Medicine 1 [year].. our ideas are modest, we don’t know much about the medical field. In Medicine 3 one would have time but it may be too late”. (First Year 4)*  *-“Some sessions, like epidemiology, were like Chinese to me: relative risk and odds ratio!! Medicine 2 students were understanding better and were more interactive than Medicine 1!”(First Year 9)*  *-“..going through the same didactic lecturing again will have us learn them for 2 weeks, just as we learnt them previously, and then forget them!” (Second Year 1)*  *-“concerning the diversity of student population, Medicine 1, 2 and 3, I think there are ways to go around this limitation with small working groups that have students from different classes. The more senior students can help the juniors grasp the info better than having a lecture that is advanced for some and trivial for others”. (Third Year 3)* |
